# Adalimumab and anti-adalimumab LISA-TRACKER immunoassays performance criteria for therapeutic drug monitoring of adalimumab-amgen biosimilar (ABP501)

**DOI:** 10.1186/s12865-021-00473-1

**Published:** 2021-12-25

**Authors:** Fabien Francois, Loubna Naimi, Xavier Roblin, Anne-Emmanuelle Berger, Stephane Paul

**Affiliations:** 1 Department of Immunology, CIC1408, GIMAP U1111/UMR5308 INSERM-UJM-UCBL-ENS de Lyon-CNRS, University Hospital of Saint-Etienne, Saint-Etienne, France; 2 Department of Gastroenterology, University Hospital of Saint-Etienne, Saint-Etienne, France

**Keywords:** Anti-TNFα, Adalimumab, Biosimilar, ABP501, AMGEVITA, HUMIRA, Drug monitoring

## Abstract

**Background:**

ABP501 is a biosimilar to Reference Adalimumab (HUMIRA®) produced by AMGEN. Adalimumab (ADA) has a marketing authorization for Crohn's disease, ulcerative colitis and other inflammatory or autoimmune diseases. The aim of this study was to evaluate the LISA-TRACKER assays developed by Theradiag (France), for the monitoring of ABP501 and anti-ABP501 antibodies in human serum.

**Results:**

68 ABP501 clinical samples were measured with the LISA TRACKER Duo Adalimumab assay. LISA TRACKER has been validated as suitable for quantification of ABP501 in human serum samples. Accuracy of the LISA-TRACKER was measured using 3 human serum matrices spiked with known levels of biosimilar, 3 levels spanning the dynamic range. Percentages of recovery were ranged from 90 to 120% for biosimilar batch1, and between 93 and 105% for biosimilar batch2. The acceptance criteria (CV < 20%) were met for intra-run (from 3.8 to 16.5%) and inter-run imprecision (from 4.4 to 13.9%) including the two batches. All results were comprised within ± 20% from results, obtained with the kit and sample unexposed in order to evaluate stability of the sample, stability of the kit and consistency of the results. In any case, but two, all percentages of inhibition were > 50% for specificity. Specificity was tested with Biosimilar spiked samples, Biosimilar with Humira® spiked samples, and clinical samples from patients treated with adalimumab biosimilar. All of these samples were spiked with polyclonal antibodies directed against Humira®. Specificity inhibition and specificity detection steps were also part of the validation parameters. Reagents made with ABP501 gave similar results than reagents made with Humira® meeting acceptance criteria.

**Conclusions:**

LISA-TRACKER ADA and anti-ADA assays are reliable for the monitoring of patients treated with ABP501.

**Supplementary Information:**

The online version contains supplementary material available at 10.1186/s12865-021-00473-1.

## Background

Reference Adalimumab (R.A.) named HUMIRA®, marketed by AbbVie, is a human monoclonal antibody to tumour necrosis factor alpha (TNFα), part of the same family as Infliximab (Remicade®, Inflectra®, Remsima®). It showed efficacy and safety for treatment of inflammatory bowel diseases (IBD) as Crohn’s disease [1] or Ulcerative colitis [1, 2], Psoriatic arthritis, Ankylosing spondylitis, Rheumatoid arthritis [3], Juvenile idiopathic arthritis [4], Hidradenitis suppurativa [5] and Uveitis [6]. This therapeutic versatility has made adalimumab the top-selling drug with global sales of $19 billion in 2019 alone [7]. From 2017, with HUMIRA’s patent expired, biosimilars started receiving marketing authorization, positive opinions of the European Medicines Agency (EMA) and the U.S. Food and Drug Administration (FDA), and new ones are currently being developed in highly regulated markets. A biosimilar is a molecule highly similar to an already licensed biologic product (referred to as the reference product) with minor differences in clinically inactive components and for which there are no clinically meaningful differences in purity, potency, and safety between the two products [8]. This is the case for, at least, six biosimilars: ABP 501 (Amgevita®, Solymbic®, Amgen, USA), GP2017 (Hefiya®, Halimatoz®, Hyrimoz®, Sandoz, Germany), BI 695501 (Cyltezo®, Boehringer Ingelheim, Germany), SB5 (Imraldi®, Biogen, South Korea), FKB327 (Hulio®, Fujifilm Kyowa Kirin, Japan), and MSB11022 (Idacio® and Kromeya®, Fresenius Kabi, Germany) [9]. ABP501 showed efficacy and safety comparable with R.A. [10], and have been approved in Europe in March 2017. The fact that therapeutic drug monitoring (TDM) of adalimumab may be associated with a lower risk of treatment failure compared with standard of care in patients [11] and that biosimilars are increasingly developed, suggests that being able to monitor biologicals as ABP501 in human serum could be interesting. LISA-TRACKER assays, based on ELISA method and developed by Theradiag (France), have already shown suitability for Infliximab biosimilars monitoring [12] and studies exposed suitability of assays for Adalimumab [13] or ABP501 [14, 15]. The purpose of this study was to evaluate, for the first time, the LISA-TRACKER assays, for the monitoring of adalimumab-amgen biosimilar and anti-adalimumab-amgen-biosimilar antibodies in human serum.

## Results

### Measure of HUMIRA®/ABP501 trough levels in spiked samples

Accuracy of the LISA-TRACKER was measured using 3 human serum matrix spiked with known levels of biosimilar, 3 levels spanning the dynamic range. For each level, the percentage of recovery was calculated (Percentage of recovery of Adalimumab assay = [Mean of the six obtained results/theoretical level of Adalimumab)] × 100).

An imprecision intra-run has been realized. Imprecision was assessed by using 3 clinical levels (low, medium, high) for 3 human matrices and for each level, 10 tests per run were performed. An imprecision inter-run has been realized. Imprecision was assessed by using 3 levels (low, medium, high) of clinical samples for 3 human matrices and for each level, on 6 independent runs, 2 tests per run were performed with LISA-TRACKER Duo Adalimumab assay.

Quantification of Adalimumab was performed according to the technical insert of LISA-TRACKER Adalimumab kits (product number LTA 005 or LTA 002). As described in the technical insert, a run performed with LTA kits was validated when the optic density (OD) of the highest standard was above 0.8 and when the result (µg/ml) of the positive control was within its target range. Then, the result of each sample could be interpreted.

#### Accuracy

Spiked samples made with batch1 gave percentages of recovery comprised between 90 and 120% and spiked samples made with batch2 gave percentages of recovery comprised between 93 and 105%. All results met the acceptance criteria (80–120%). ABP501 spiked samples were quantified with LISA-TRACKER Duo Adalimumab assay (Table 1). Quantification of ABP501 was not affected by serum matrix. Similar results were obtained with Humira® during the development of LISA-TRACKER Duo Adalimumab assay.

**Table 1:** Determination of the mean recovery in drug measurement obtained for the two batches of ABP501

Spiked samples (target); µg/ml	Acceptance criteria (µg/ml)		Matrix 1 (M1)		Matrix 2 (M2)		Matrix 3 (M3)		Total	
	Low	High	µg/ml	Mean (µg/ml)	µg/ml	Mean (µg/ml)	µg/ml	Mean (µg/ml)	µg/ml	% of recovery (%)
*ADALIMUMAB from ABP501-Batch1*										
1	0.8	1.2	1.3	1.3	1.2	1.3	0.9	1.1	1.20	120
			1.3		1.3		1.2			
4	3.2	4.8	2.5	3.3	3.5	3.7	3.7	3.9	3.6	90
			4.1		3.8		4.1			
12	9.6	14.4	13.0	13.2	8.9	10.9	10.8	12.7	12.2	102
			13.3		129		14.5			
*ADALIMUMAB from ABP501-Batch2*										
1	0.8	1.2	1.0	1.0	0.7	0.8	1.0	1.1	0.9	93
			0.9		0.9		1.1			
4	3.2	4.8	4.2	4.4	3.7	4.1	3.6	4.3	4.2	105
			4.5		4.4		4.9			
12	9.6	14.4	13.4	11.8	10.1	11.6	12.8	13.6	12.3	103
			10.2		13.0		14.4			

Accuracy of the LISA-TRACKER was measured using 3 human serum matrix spiked with known levels of biosimilar (ABP501-Batch1 or ABP501-Batch2), 3 levels spanning the dynamic range: 1, 4 and 12 µg/ml. Quantification with LISA-TRACKER adalimumab assay and the percentages of recovery were calculated for the ABP501 batch1 and the ABP501 batch2. All the quantifications were established by LISA-TRACKER adalimumab assay

Acceptance criteria: 80% < N < 120%

#### Intra-run and inter-run imprecision

For the imprecision intra-run, the CV ranged from 4.1 to 11.1% for samples made with biosimilar batch1 and ranged from 3.8 to 16.5% for samples made with biosimilar batch2 (Table 2a). For the imprecision inter-run, the CV ranged from 4.4 to 13.9% for samples made with biosimilar batch1 and 4.5 to 13.0% for samples made with biosimilar batch2 (Table 2b). The acceptance criteria (CV < 20%) were met.

**Table 2:** Determination of the imprecision for ABP501 quantification

	ADALIMUMAB from ABP501-Batch1 (µg/ml)									ADALIMUMAB from ABP501-Batch2 (µg/ml)								
	Matrix 1 (M1)			Matrix 2 (M2)			Matrix 3 (M3)			Matrix 1 (M1)			Matrix 2 (M2)			Matrix 3 (M3)		
	Low	Medium	High	Low	Medium	High	Low	Medium	High	Low	Medium	High	Low	Medium	High	Low	Medium	High
*(a) Intra-run: 1 run for each batch, 10 tests per level for each matrix*																		
Mean (µg/ml)	1.5	5.0	13.6	1.6	4.5	15.9	1.4	4.3	15.1	1.2	3.9	14.6	0.6	5.0	13.6	1.0	3.6	13.3
CV (%)	4.9	7.6	6.8	6.9	5.4	4.1	6.0	6.4	11.1	14.4	3.8	7.8	16.5	12.8	4.4	4.5	4.5	5.3
*(b) Inter-run: 6 runs including the 2 batches, 2 tests per level for each matrix*																		
Mean (µg/ml)	1.3	4.2	12.4	1.1	4.0	13.1	1.1	3.8	11.6	1.1	4.1	12.3	1.07	4.2	11.6	1.0	4.0	11.6
CV (%)	9.7	5.2	4.4	12.2	8.9	9.2	11.2	13.9	4.9	9.6	9.3	6.6	9.1	13.0	4.5	8.0	8.9	6.7

Imprecision was assessed by using clinical 3 levels (low, medium, high) for 3 human matrix and for each level. Tests were performed with LISA-TRACKER adalimumab assay for the ABP501 batch#1 and batch#2. The coefficients of determination were determined. All the quantifications were established by LISA-TRACKER adalimumab assay

(a) For Intra-run, 1 run for each batch, 10 tests per level for each matrix were performed

(b) For Inter-run, 6 runs including the 2 batches, 2 tests per level for each matrix were performed

Acceptance criteria: CV < 20%

Low intra-run and inter-run imprecisions were reached with LISA-TRACKER Duo Adalimumab assay for the quantification of ABP501.

### Measure of HUMIRA®/AB501 through levels in clinical samples

Clinical samples with detectable level of Adalimumab (Humira®) were spiked with Humira® or ABP501. Thus, Adalimumab (Humira® or ABP501) were added into the clinical samples in order to increase the level of Adalimumab of 4 µg/ml. All samples from the 3 preparations (3 × 30 spiked clinical samples) were quantified with LISA-TRACKER Adalimumab kit (LTI 002, batch: 1847). The results obtained with ABP501 spiked clinical samples were compared to the results obtained with Humira® spiked clinical samples. Furthermore, the same type of tests was launched with clinical samples with detectable level of adalimumab biosimilar. Thus, ABP501 clinical samples were spiked with Humira® in order to increase the level of 4 µg/ml; spiked samples were quantified and compared to the unspiked samples.

#### ABP501 levels in patients

There were 30 clinical samples with detectable level of Humira®. All results from clinical samples spiked with ABP501 were within ± 20% of the expected value [levels of Adalimumab from clinical samples spiked with Adalimumab (Humira®)] (Additional file 1: Table S1a).

**Fig. 1:**
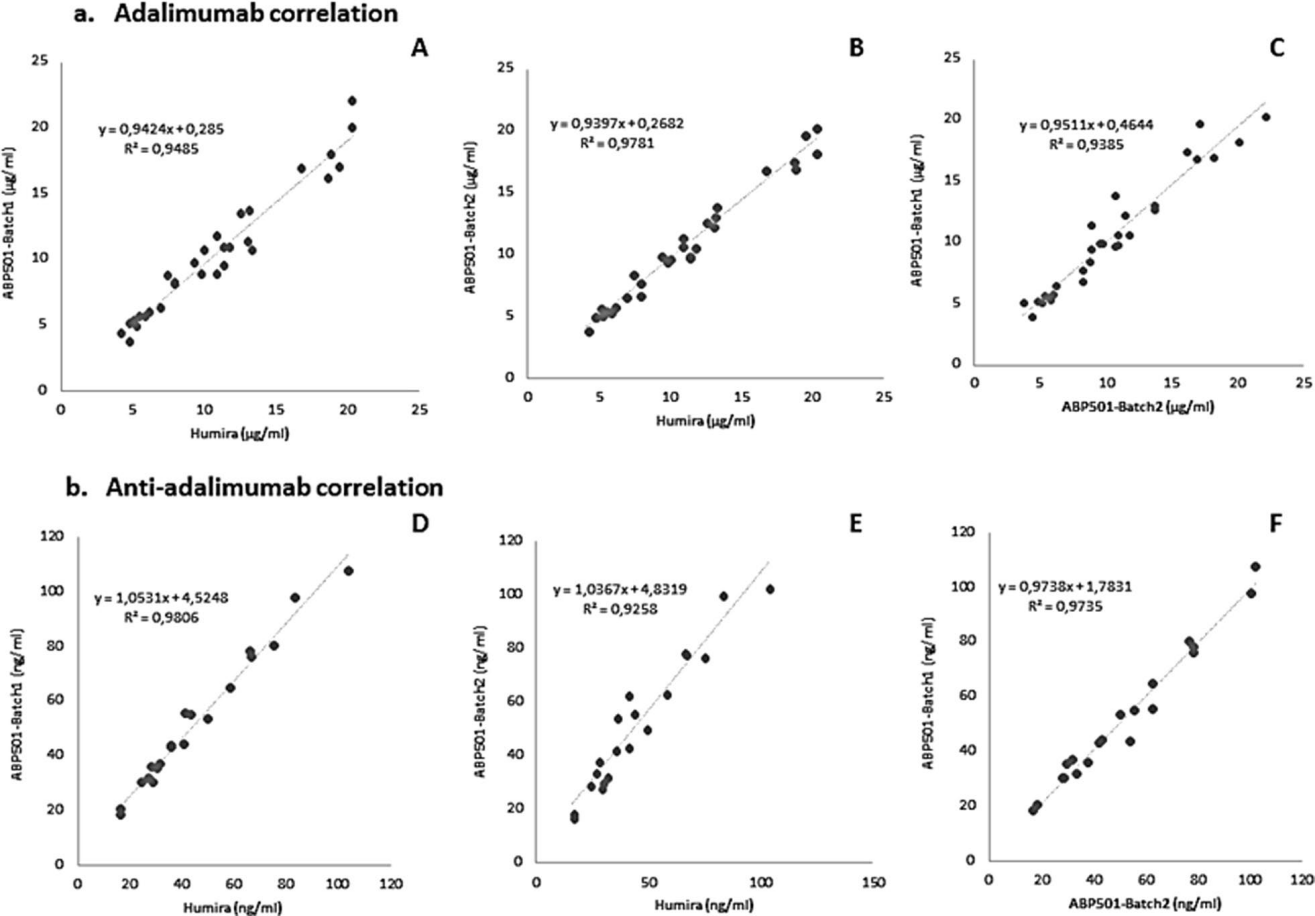
Measurement of levels of adalimumab (ABP501/Humira®) and anti-adalimumab (Anti-ABP501/Anti-Humira®) in patients or spiked samples according to the Adalimumab standard curve. The coefficient of determination (R^2^) and the slope were calculated for the 3 different combinations. **a** Adalimumab correlation: ABP501-batch1 versus Humira® (R^2^ = 0.95; slope: 0.94) (**A**), ABP501-batch2 versus Humira® (R^2^ = 0.98; slope: 0.94) (**B**), ABP501-batch1 versus ABP501-batch2 (R^2^ = 0.94; slope: 0.95) (**C**). **b** Anti-adalimumab correlation: ABP501-batch1 versus Humira® (R^2^ = 0.98; slope: 1.05) (**D**), ABP501-batch2 versus Humira® (R^2^ = 0.93; slope: 1.04) (**E**), ABP501-batch1 versus ABP501-batch2 (R^2^ = 0.97; slope: 0.97) (**F**). All the quantifications were measured with the LISA-TRACKER Duo Adalimumab assay

The coefficient of determination (R^2^) and the slope were calculated for the 3 different preparations. ABP501-batch1 against Humira® (R^2^ = 0.95; slope: 0.94), ABP501-batch2 against Humira® (R^2^ = 0.98; slope: 0.94), ABP501-batch1 against ABP501-batch2 (R^2^ = 0.94; slope: 0.95) (Fig. 1a). Thus, all the coefficients of determination and Slopes met the acceptance criteria (R^2^ > 0.90 and a slope comprised between 0.9 and 1.1).

Detection of ABP501 trough levels in the patients treated with ABP501 (34 clinical samples, ID: ABP501-1 to ABP501-34) with the LISA-TRACKER Adalimumab assay was compliant: all ABP501 positive samples were in the expected range after the addition of 4 µg/ml of R.A. (Additional file 1: Table S1b).

Therefore LISA-TRACKER Duo Adalimumab assay detect efficiently ABP501 trough levels as the R.A. in serum without any interference.

### Specificity assessment: adalimumab assay

ABP501 spiked samples inhibition assays were performed with LISA-TRACKER Adalimumab kit (product number: LTA 002, batch: 1847). In order to confirm the positivity of ABP501 spiked samples and the positivity of clinical samples, polyclonal antibodies directed against Humira® were added to the kit’s dilution buffer: poly-anti-ada-buffer. On one hand, ABP501 spiked samples (ABP501 spiked samples and ABP501 + Humira® spiked samples) and clinical samples were diluted with poly-anti-ada-buffer, and on the other hand they were diluted with the kit’s dilution buffer. Quantification of the samples was performed after 60 min of incubation at room temperature. For each sample, the percentage of inhibition was calculated. (Percentage of inhibition of Adalimumab assay = [1 − (level of Adalimumab from sample diluted with poly-anti-ada-buffer/level of Adalimumab from sample diluted with sample dilution buffer)] × 100).

Spiked samples (ABP501 spiked samples and ABP501 + Adalimumab (Humira®) spiked samples) and clinical samples with a percentage of inhibition greater than 50% were considered positive (detectable level) for ABP501.

#### Specificity

For ABP501 spiked samples, the levels of Adalimumab were below the limit of quantification when the kit’s dilution buffer spiked with polyclonal was used. All percentages of inhibition were at least above 77%. For ABP501 + Adalimumab spiked samples made with Humira® in addition with ABP501, the levels of Adalimumab were below the limit of quantification when the kit’s dilution buffer spiked with the polyclonal antibodies was used (Additional file 1: Table S2a). For ABP501 clinical samples (ID: ABP501-35 to ABP501-68), the levels of Adalimumab were below the limit of quantification when the kit’s dilution buffer spiked with the polyclonal antibodies was used. All percentages of inhibition were at least above 92% (Additional file 1: Table S2b).

Acceptance criteria (percentage of inhibition > 50%) were met. ABP501 spiked samples, ABP501 + Adalimumab spiked samples and clinical samples gave similar results.

Anti-Adalimumab antibodies generated with Humira® have the capacity to block Humira® and ABP501. LISA-TRACKER Duo Adalimumab assay should be able to detect ABP501 as it does for Humira®.

### Measure of anti-adalimumab in clinical samples: inhibition assay

Clinical samples with detectable level of anti-adalimumab antibodies were diluted with the kit’s dilution buffer previously spiked with Adalimumab (Humira® or ABP501 were added to kit’s dilution buffer in order to prepare 2 types of ada-buffer). In addition, the clinical samples were diluted with the kit’s dilution buffer. The 3 preparations (2 preparations made with Adalimumab and 1 preparation made without Adalimumab) were incubated 60 min at room temperature and quantified with LISA-TRACKER Anti-Adalimumab kit (product number LTA 003, batch: 1844). For each sample, the percentage of inhibition was calculated (percentage of inhibition of anti-adalimumab assay = [1 − (level of anti-adalimumab antibodies from sample diluted with ada-buffer/level of anti-adalimumab antibodies from sample diluted with kit’s dilution buffer)] × 100). The capacity of ABP501 to block the detection of ATA was measured.

Clinical samples with a percentage of inhibition greater than 50% were considered to be inhibited by Adalimumab.

#### Adalimumab and ATA inhibition assay

There were 31 clinical samples (ID: AADA1 to AADA31) with detectable levels of Anti-Adalimumab antibodies.

Samples with high levels of anti-adalimumab antibodies gave high percentages of inhibition because they were well above the LLOQ. In any case, all percentages were above 50% and both batches of ABP501 and Adalimumab gave similar results. Acceptance criteria were met (Additional file 1: Table S3).

ABP501 is able to block anti-adalimumab antibodies from patients treated with Humira®.

### Measure of specificity detection step in spiked clinical samples

Clinical samples with detectable level of anti-adalimumab antibodies were quantified with LISA-TRACKER anti-adalimumab kit (product number: LTA 003, batch: 1849). In order to confirm the capacity of ABP501 to block antibodies directed against Humira®, detection step was performed with or without the addition of ABP501 into the detection reagent (biotinylated Humira®) used for the detection step of Anti-Adalimumab antibodies during the assay). For each sample, the percentage of inhibition was calculated (percentage of inhibition of Anti-Adalimumab assay = [1 − (level of Anti-Adalimumab antibodies in the presence of ABP501 into the detection reagent/level of Anti-Adalimumab antibodies)] × 100).

Clinical samples with a percentage of inhibition greater than 50% were considered to be inhibited by ABP501.

#### Specificity detection step

There were 38 clinical samples (ID: AADA32 to AADA69) with detectable levels of Anti-Adalimumab antibodies. Samples with low levels [around 2 × limit of quantification (LLOQ)] of Anti-Adalimumab antibodies gave percentages of inhibition near 50% because they were near the limit of quantification (10 ng/ml). Samples with high levels of Anti-Adalimumab antibodies gave high percentages of inhibition because they were well above the LLOQ. In any case, but two, all percentages were above 50%. Both batches of ABP501 gave similar results: acceptance criteria were met (Additional file 1: Table S4).

ABP501 is able to compete with biotinylated Humira®. Anti-Adalimumab antibodies induced by Humira® were able to detect ABP501 during the detection step. LISA-TRACKER Duo Adalimumab assay should be able to detect anti-adalimumab antibodies induced by ABP501.

### Measure of anti-adalimumab/anti-ABP501 in spiked samples

Clinical samples with detectable level of Anti-Adalimumab antibodies were quantified with the 3 pairs of reagents made with the 3 types of raw materials (pair1: Humira® coated microplate and biotinylated Humira®; pair2: ABP501-batch1 coated microplate and biotinylated ABP501-batch1; pair3: ABP501-batch2 coated microplate and biotinylated ABP501-batch2). Results from the 3 pairs were compared.

#### Measurement of levels of anti-adalimumab in patient and spiked samples according to the anti-adalimumab standard curve

There were 20 clinical samples (ID: AADA70 to AADA89) with detectable levels of Anti-Adalimumab antibodies. The coefficient of determination (R^2^) and the slope were calculated for the 3 different combinations of reagents. ABP501-batch1 versus Humira® (R^2^ = 0.98; slope: 1.05), ABP501-batch2 versus Humira® (R^2^ = 0.93; slope: 1.04), ABP501-batch1 versus ABP501-batch2 (R^2^ = 0.97; slope: 0.97) (Fig. 1b). Thus, all the coefficients of determination and Slopes met the acceptance criteria (R^2^ > 0.90 and a slope comprised between 0.9 and 1.1).

Reagents made with ABP501 give the same performances as reagents made with Humira® for the detection of Anti-Adalimumab antibodies (from patients treated with Humira®). This demonstrates the similarity of ABP501 and Humira® towards Anti-Adalimumab antibodies. LISA-TRACKER Anti-Adalimumab assay should be able to detect Anti-Adalimumab antibodies induced by ABP501 as it does for Anti-Adalimumab antibodies induced by Humira®.

### Measure of kit and sample stability

In order to evaluate the kit’s stability, LISA-TRACKER Duo Adalimumab kit was stored under stress thermic condition (7 days at + 37 °C). Then, ABP501 spiked samples were tested with this “stressed” kit. Results were compared to the results obtained with the unexposed kit (stored between + 2 and + 8 °C).

For specimen’s stability, spiked samples from patients treated with adalimumab biosimilar were stored in different conditions until quantification: at − 20 °C (unexposed samples), 7 days between + 2 and + 8 °C (+ 4 °C storage condition), 3 days between + 18 and + 24 °C (room temperature (RT) storage condition), and 5 freeze/thaw cycles undergone.

#### Kit’s stability

The percentages of variation (results from the stressed kit compared to the unexposed kit) were comprised between − 11 and 17% for spiked samples made with ABP501-batch1, and between − 3 and 18% for spiked samples made with ABP501-batch2. All results were within ± 20% from the unexposed kit: acceptance criteria were met. LISA-TRACKER Duo Adalimumab assay is robust. Quantification of ABP501 was not disrupted even if the kit was stored over a long period of elevated temperature (delivery, storage, etc.) (Additional file 1: Table S5a).

#### Specimen’s stability

For all storage conditions (+ 4 °C, RT or freeze/thaw cycles), all samples gave levels of Adalimumab comprised within ± 20% compared to the unexposed samples. ABP501 serum samples can be stored, 7 days at + 4 °C, or 3 days at RT, or can undergo up to 5 freeze/thaw cycles, before being quantified with LISA-TRACKER Duo Adalimumab assay (Additional file 1: Table S5b).

All measurements done, reagents made with ABP501 gave similar results than reagents made with Humira® meeting acceptance criteria (Table 3).

**Table 3:** Summary of the results found

Parameters			Description	Acceptance criteria	Results		Tables
**Adalimumab**							
*Accuracy*		Samples spiked with ABP501 in 3 matrices	2 runs including the 2 batches. 1 test per level for each matrix	80% < N < 120%	Batch 1	90–120%	Table 1
Imprecision	Intra-run				Batch 2	93–105%	
			1 run for each batch. 10 tests per level for each matrix	CV < 20%	Batch 1	4.1–11.1%	Table 2a
					Batch 2	3.8–16.5%	
	Inter-run		6 runs including the 2 batches. 2 tests per level for each matrix		Batch 1	4.4–13.9%	Table 2b
					Batch 2	4.5–13.0%	
Humira/AB501 levels in clinical samples	Humira clinical samples spiked with ABP501		30 samples	In a range of ± 20%	Within the range		Additional file 1: Table S1a
	ABP501 clinical samples spiked with Humira		34 samples				Additional file 1: Table S1b
*Correlation*		3 runs × 30 samples spiked with: Run 1: ABP501 batch 1 Run 2: ABP501 batch 2 Run 3: Humira	R^2^ > 0.90 0.9 < Slope < 1.1	Batch1 versus Humira	R^2^ = 0.95	Slope = 0.94	Figure 1a
				Batch2 versus Humira	R^2^ = 0.98	Slope = 0.94	
				Batch1 versus Batch2	R^2^ = 0.94	Slope = 0.95	
**Anti-Adalimumab**							
Inhibition	Samples spiked with ABP501 or ABP501 + Humira. then with Anti-Ada antibodies	31 samples	% of inhibition > 50%		> 77%		Additional file 1: Table S2a
	ABP501 clinical samples spiked with Anti-Ada antibodies	34 samples			> 92%		Additional file 1: Table S2b
	Clinical samples with Anti-Ada antibodies spiked with Adalimumab (ABP501 or Humira)	31 samples			> 62%		Additional file 1: Table S3
Detection step	Clinical samples diluted with Anti-Ada antibodies spiked with ABP501	38 samples			51–89% Except two with Anti-ADA levels near the LLOQ		Additional file 1: Table S4
*Correlation*		3 runs × 20 samples with Anti-Ada antibodies spiked with: Run 1: ABP501 batch 1 Run 2: ABP501 batch 2 Run 3: Humira	R^2^ > 0.90 0.9 < Slope < 1.1	Batch1 versus Humira	R^2^ = 0.98	Slope = 1.05	Figure 1b
				Batch2 versus Humira	R^2^ = 0.93	Slope = 1.04	
				Batch1 versus Batch2	R^2^ = 0.97	Slope = 0.97	
Kit’s stability	Samples spiked with ABP501 in different storage conditions for each run	2 kits. 1 run for each including the 2 bacthes. 2 tests per level	In a range of ± 20%		Within the range		Additional file 1: Table S5
Specimen’s stability		4 runs including the 2 batches. 2 tests per level for each storage condition					

## Discussion

In this study, by analysing the results obtained, quantification of ABP501 was not affected by serum matrix. Low intra-run and inter-run imprecisions were reached with LISA-TRACKER Duo Adalimumab assay for the quantification of ABP501. Similar results for the two batches were found. LISA-TRACKER Duo Adalimumab assay should be able to detect efficiently ABP501 as it does for R.A. in serum without any interference, and detect Anti-Adalimumab antibodies induced by ABP501, as it does for Anti-Adalimumab antibodies induced by Humira®. The two results out of our acceptance criteria for specificity detection step cannot question the last point because the initial level of ATA was near the LLOQ, underestimating the percentage of inhibition. Regarding the fact that quantification of ABP501 was not disrupted even if the kit was stored over a long period of elevated temperature (delivery, storage, etc.), it expresses the robustness of the collected results. These performance criteria, including reproducibility, attest to the suitability of LISA-TRACKER Duo Adalimumab assay for ABP501 and anti-ABP501 measurement. Therapeutic drug monitoring of monoclonal antibodies allows clinicians to more safely, effectively, and efficiently use medications [16]. A recent retrospective cohort study emphasized the importance of measuring adalimumab serum levels early, which may guide dose optimization and prevent immunogenicity with associated treatment failure [17]. Other studies showed that TDM limits unnecessary dose escalation and provides appropriate treatment strategy without compromising clinical outcomes [18]. Researches have been made using LISA-TRACKER immunoassays for different anti-TNFα as Infliximab, Adalimumab, Etanercept [19], Certolizumab and Golimumab [20], but a few presented results about biosimilars. The increasing development of new biosimilars as ABP501 and the opportunity to switch from the originator adalimumab to a biosimilar compound without affecting treatment efficacy [21–23], leads to the fact that these biologicals actually represent great potential, allowing patients greater access to monoclonal antibodies [24] and will have a more important role for TDM in the future. To this end, this study suggests a new option for drug monitoring in order to guide the management of patients with loss of response to ABP501 as it has been described for R.A in Crohn’s disease [25].

## Conclusions

LISA-TRACKER Duo Adalimumab kit (LTA 005), LISA-TRACKER Adalimumab kits (LTA 002-48, LTA 002-96) and LISA-TRACKER anti-Adalimumab kits (LTA 003-48, LTA 003-96) are suitable for the monitoring of patients treated with ABP501. Our study shows an acceptable correlation of dosages between the ATRA and Anti-ABP501, as well as detection of AB501. Thus, the monitoring of ABP501 will be useful for the follow-up of patients with inflammatory diseases, for the research and for the therapeutic optimization.

## Methods

### Reagents

ABP501 batches were provided by AMGEN and HUMIRA®, marketed by AbbVie have been used in this study in order to spike samples.

These immunoassays have been manufactured by THERADIAG that provided LISA-TRACKER Duo Adalimumab (product number: LTA 005) and reagents including:

- Biotinylated antibody vials
- HRP labelled Streptavidin
- «Positive control—Adalimumab», (μg/mL)
- «Positive control—anti-Adalimumab», (ng/mL)
- Phosphate-Tween Buffer pH 7,2 (10x), Substrate (TMB)
- Stop solution—H_2_SO_4_ (0.25 N)
- Strips of individual breakaway blue wells coated with human TNFα
- Strips of individual breakaway black wells coated with Adalimumab
- 5 vials of « Adalimumab» Standards, (μg/mL)
- 5 vials of « anti-Adalimumab» Standards, (ng/mL)

### Experiment protocol

The assay protocol has been conducted in order to evaluate the performance criteria of LISA TRACKER immunoassays for adalimumab and anti-adalimumab biosimilar antibodies.

According to the results, the monitoring of the patients treated with ABP501 could be interesting, using the kit developed by Theradiag and based on ELISA method. To this end, 30 clinical samples from patients treated with reference Adalimumab (Humira®), and 89 serum samples from patients treated with Humira® with detectable levels of anti-adalimumab antibodies were collected from France territory by Theradiag without specific clinical criteria. Serum samples have been selected in order to have measurable levels of Adalimumab or antibodies directed to it. Then, 68 serum samples provided by AMGEN, from patients treated with ABP501, were used. All clinical samples were analyzed anonymously, collected during routine clinical practice and were provided without clinical information.

Preparations of ABP501 spiked samples were made with 3 human serum matrices (two from individual healthy donors and one from a pool of healthy donors: M1, M2 and M3 respectively) in addition to ABP501 solutions spiked to reach 3 levels of drug: 1, 4 and 12 µg/ml (low, medium, high respectively).

LISA-TRACKER Duo Adalimumab, is an enzyme linked immunoassay (ELISA) for the quantitative determination of Adalimumab (anti-TNFα) and anti-Adalimumab antibodies in human serum samples. These tests can be separately or simultaneously done by following the standardized assay protocol.

Initially, the TNFα and the antibodies are coated onto a polystyrene microtiter plate (6 strips of 8 wells). Samples were diluted with dilution and wash buffer (TDL): 5 μl sample added to 1 ml TDL (dilution of 1/201) for Adalimumab assay, and 130 μl sample added to 130 μl TDL (dilution of 1/2) for anti-Adalimumab assay. First, the diluted samples were incubated, at least half an hour, at room temperature (+ 18 °C/+ 25 °C). Then, 100 µl of diluted sample were added to the coated well, which allows to bind: TNFα coated well for Adalimumab assay, antibody coated well for anti-Adalimumab assay. After 20 min at room temperature, unbound antibodies or proteins were removed by washing. Each wash step involved washing 3 times with 300μL of TDL buffer. Then, 100 µl of biotinylated polyclonal rabbit anti-Adalimumab antibody or Adalimumab was added. A new wash step has been performed in order to eliminate any excess of unbound molecules after 15 min of incubation. Later, 100 µl of horseradish peroxidase labelled streptavidin was added. The streptavidin binds to the complex formed with biotinylated anti-ADA antibodies or Adalimumab. After 15 min of incubation, the wells were washed again to eliminate any excess of conjugate. The bound enzyme was revealed by addition of 100 µl of substrate TMB (3,3′,5,5′ tetramethylbenzidin). The colour intensity was proportional to the amount of Adalimumab or antibodies. Adding 100 µl of H_2_SO_4_ allowed to stop the enzymatic reaction. After stopping the reaction by H_2_SO_4_, the optical density was read by a spectrophotometer at 450 nm. A range of calibration allowed to define the quantity of Adalimumab or antibodies of each sample.

Reagents and micro-wells were stored at + 2 °C/+ 8 °C in their own package. To avoid any non-specific binding, serum samples which had been frozen or which were cloudy, have also been centrifuged and filtered. All reagents have been unpacked, at least half an hour before starting the test, in order to let them warm at room temperature.

Accuracy, standard curves comparison, intra-run imprecision, inter-run imprecision, correlation, kit’s stability, specificity inhibition and specificity detection step were part of the LISA-TRACKER Duo Adalimumab assay validation parameters. Lower Limit Of Quantification (LLOQ) of ADALIMUMAB was 0.3 µg/ml and LLOQ of Anti-ADALIMUMAB assay was 10 ng/ml.

Acceptance criteria, based on FDA guideline “Guidance for Industry—Bioanalytical method validation-ligand binding assay-Sept2013” and EMA guideline “Guideline on bioanalytical method validation-ligand binding assays-21 July 2011”, were similar to the criteria used for the validation of LISA-TRACKER assays. The study was considered exempt by the Ethics Committee Board of Saint-Etienne Hospital and the Centre National Informatique et Liberté (CNIL) (Number: 1849323).

## Supplementary Information

**Additional file 1.** Supplementary datas.

## Data Availability

All the datas and material are available upon request.

## Abbreviations

ATA: Antibodies to adalimumab
CV: Coefficient of variation
ELISA: Enzyme-linked immunosorbent assay
FDA: Food and drug administration
ADA: Adalimumab
OD: Optic density
R.A.: Reference adalimumab
ATRA: Antibodies to reference adalimumab
TNFα: Tumour necrosis factor alpha
LLOQ: Lower limit of quantification
TDM: Therapeutic drug monitoring
