# Supplementary material for: Adalimumab and anti-adalimumab LISA-TRACKER immunoassays performance criteria for therapeutic drug monitoring of adalimumab-amgen biosimilar (ABP501)
Source: BMC Immunol. 2021 Dec 25;22:81. doi: 10.1186/s12865-021-00473-1 (PMC8710016; doi:10.1186/s12865-021-00473-1)
Supplement: Supplementary file 1 — Additional file 1. Supplementary datas. [file 12865_2021_473_MOESM1_ESM.docx]

| **a.** |  |  |  |  |  |
| --- | --- | --- | --- | --- | --- |
|  | **ADALIMUMAB (µg/ml)** | | | | |
| **Clinical Samples Spiked with :** | **ADALIMUMAB (Humira)** | ***Acc. Criteria.*** | | **ABP501-Batch1** | **ABP501-Batch2** |
|  |  | ***Low*** | ***High*** |  |  |
| **1** | 5,1 | *4,1* | *6,1* | 5,4 | 5,6 |
| **2** | 13,2 | *10,6* | *15,8* | 13,7 | 13,0 |
| **3** | 11,8 | *9,4* | *14,2* | 10,9 | 10,5 |
| **4** | 6,1 | *4,9* | *7,3* | 6,0 | 5,7 |
| **5** | 20,3 | *16,2* | *24,4* | 22,1 | 20,3 |
| **6** | 9,4 | *7,5* | *11,3* | 9,8 | 9,9 |
| **7** | 7,9 | *6,3* | *9,5* | 8,3 | 6,7 |
| **8** | 13,1 | *10,5* | *15,7* | 11,4 | 12,2 |
| **9** | 16,8 | *13,4* | *20,2* | 16,9 | 16,8 |
| **10** | 5,5 | *4,4* | *6,6* | 5,7 | 5,5 |
| **11** | 6,9 | *5,5* | *8,3* | 6,3 | 6,5 |
| **12** | 10,9 | *8,7* | *13,1* | 8,9 | 11,4 |
| **13** | 12,6 | *10,1* | *15,1* | 13,6 | 12,6 |
| **14** | 5,2 | *4,2* | *6,2* | 4,9 | 5,1 |
| **15** | 7,4 | *5,9* | *8,9* | 8,8 | 8,4 |
| **16** | 7,9 | *6,3* | *9,5* | 8,2 | 7,7 |
| **17** | 10,9 | *8,7* | *13,1* | 11,8 | 10,6 |
| **18** | 4,8 | *3,8* | *5,8* | 5,1 | 5,0 |
| **19** | 10,0 | *8,0* | *12,0* | 10,7 | 9,7 |
| **20** | 9,8 | *7,8* | *11,8* | 8,9 | 9,4 |
| **21** | 11,4 | *9,1* | *13,7* | 9,5 | 9,9 |
| **22** | 4,2 | *3,4* | *5,0* | 4,4 | 3,9 |
| **23** | 5,9 | *4,7* | *7,1* | 5,7 | 5,3 |
| **24** | 11,4 | *9,1* | *13,7* | 10,9 | 9,8 |
| **25** | 18,8 | *15,0* | *22,6* | 18,1 | 16,9 |
| **26** | 13,3 | *10,6* | *16,0* | 10,7 | 13,8 |
| **27** | 4,8 | *3,8* | *5,8* | 3,8 | 5,0 |
| **28** | 19,5 | *15,6* | *23,4* | 17,1 | 19,6 |
| **29** | 20,3 | *16,2* | *24,4* | 20,0 | 18,2 |
| **30** | 18,7 | *15,0* | *22,4* | 16,2 | 17,4 |

| **b.** |  |  |  |  |  |
| --- | --- | --- | --- | --- | --- |
|  | **ADALIMUMAB (µg/ml)** | | | | |
| **Clinical Samples:** | **unspiked** | **+4µg/ml** | ***Acc. Criteria.*** | | **ADALIMUMAB (HUMIRA)** |
|  |  |  | ***Low*** | ***High*** |  |
| **ABP501-1** | 8,6 | 12,6 | *10,1* | *15,1* | 14,3 |
| **ABP501-2** | 9,6 | 13,6 | *10,9* | *16,3* | 14,7 |
| **ABP501-3** | 9,7 | 13,7 | *11,0* | *16,4* | 12,4 |
| **ABP501-4** | 6,8 | 10,8 | *8,6* | *13,0* | 12,3 |
| **ABP501-5** | 9,0 | 13,0 | *10,4* | *15,6* | 15,3 |
| **ABP501-6** | 11,9 | 15,9 | *12,7* | *19,1* | 15,4 |
| **ABP501-7** | 10,0 | 14,0 | *11,2* | *16,8* | 12,0 |
| **ABP501-8** | 8,1 | 12,1 | *9,7* | *14,5* | 10,8 |
| **ABP501-9** | 6,9 | 10,9 | *8,7* | *13,1* | 11,9 |
| **ABP501-10** | 7,5 | 11,5 | *9,2* | *13,8* | 10,6 |
| **ABP501-11** | 6,7 | 10,7 | *8,6* | *12,8* | 9,7 |
| **ABP501-12** | 9,6 | 13,6 | *10,9* | *16,3* | 12,5 |
| **ABP501-13** | 14,0 | 18,0 | *14,4* | *21,6* | 15,8 |
| **ABP501-14** | 4,7 | 8,7 | *7,0* | *10,4* | 7,9 |
| **ABP501-15** | 11,5 | 15,5 | *12,4* | *18,6* | 15,8 |
| **ABP501-16** | 6,8 | 10,8 | *8,6* | *13,0* | 12,6 |
| **ABP501-17** | 16,3 | 20,3 | *16,2* | *24,4* | 16,3 |
| **ABP501-18** | 9,8 | 13,8 | *11,0* | *16,6* | 13,3 |
| **ABP501-19** | 12,2 | 16,2 | *13,0* | *19,4* | 15,2 |
| **ABP501-20** | 18,9 | 22,9 | *18,3* | *27,5* | 24,6 |
| **ABP501-21** | 5,9 | 9,9 | *7,9* | *11,9* | 9,4 |
| **ABP501-22** | 4,0 | 8,0 | *6,4* | *9,6* | 7,0 |
| **ABP501-23** | 4,3 | 8,3 | *6,6* | *10,0* | 8,5 |
| **ABP501-24** | 9,8 | 13,8 | *11,0* | *16,6* | 14,8 |
| **ABP501-25** | 5,4 | 9,4 | *7,5* | *11,3* | 8,6 |
| **ABP501-26** | 5,8 | 9,8 | *7,8* | *11,8* | 9,6 |
| **ABP501-27** | 11,4 | 15,4 | *12,3* | *18,5* | 17,8 |
| **ABP501-28** | 8,3 | 12,3 | *9,8* | *14,8* | 14,6 |
| **ABP501-29** | 13,4 | 17,4 | *13,9* | *20,9* | 17,4 |
| **ABP501-30** | 5,8 | 9,8 | *7,8* | *11,8* | 10,0 |
| **ABP501-31** | 11,9 | 15,9 | *12,7* | *19,1* | 15,8 |
| **ABP501-32** | 4,3 | 8,3 | *6,6* | *10,0* | 8,3 |
| **ABP501-33** | 4,2 | 8,2 | *6,6* | *9,8* | 8,2 |
| **ABP501-34** | 6,1 | 10,1 | *8,1* | *12,1* | 10,4 |

**Additional file 1: Table S1. Humira/AB501 levels in clinical samples.**

a) Clinical samples with detectable level of Adalimumab (Humira®) were spiked with Adalimumab (Humira®) or ABP501. Thus, Adalimumab (Humira® or ABP501) were added into the clinical samples in order to increase the level of Adalimumab of 4µg/ml. All samples from the 3 preparations (3 x 30 “spiked clinical samples”) were quantified with LISA-TRACKER Adalimumab kit.

b) The same type of tests was launched with clinical samples with detectable level of adalimumab biosimilar. Thus, ABP501 clinical samples were spiked with Humira® in order to increase the level of 4µg/ml; spiked samples were quantified and compared to the unspiked samples.

**Acceptance criteria : +/-20%**

| **b.** | | | |
| --- | --- | --- | --- |
| **SAMPLES** | µg/ml | | % of inhibition*** |
|  | ***without polyclonal Ab*** | ***with polyclonal Ab*** |  |
| **ABP501-35** | 5,8 | <0,3 | 95% |
| **ABP501-36** | 6,0 | <0,3 | 95% |
| **ABP501-37** | 6,4 | <0,3 | 95% |
| **ABP501-38** | 5,7 | <0,3 | 95% |
| **ABP501-39** | 7,3 | <0,3 | 96% |
| **ABP501-40** | 8,0 | <0,3 | 96% |
| **ABP501-41** | 4,4 | <0,3 | 93% |
| **ABP501-42** | 6,3 | <0,3 | 95% |
| **ABP501-43** | 8,5 | <0,3 | 96% |
| **ABP501-44** | 7,2 | <0,3 | 96% |
| **ABP501-45** | 7,8 | <0,3 | 96% |
| **ABP501-46** | 6,0 | <0,3 | 95% |
| **ABP501-47** | 5,3 | <0,3 | 94% |
| **ABP501-48** | 8,8 | <0,3 | 97% |
| **ABP501-49** | 4,3 | <0,3 | 93% |
| **ABP501-50** | 10,9 | <0,3 | 97% |
| **ABP501-51** | 8,5 | <0,3 | 96% |
| **ABP501-52** | 14,0 | <0,3 | 98% |
| **ABP501-53** | 8,9 | <0,3 | 97% |
| **ABP501-54** | 10,0 | <0,3 | 97% |
| **ABP501-55** | 23,4 | <0,3 | 99% |
| **ABP501-56** | 5,3 | <0,3 | 94% |
| **ABP501-57** | 11,8 | <0,3 | 97% |
| **ABP501-58** | 4,8 | <0,3 | 94% |
| **ABP501-59** | 7,0 | <0,3 | 96% |
| **ABP501-60** | 13,8 | <0,3 | 98% |
| **ABP501-61** | 13,7 | <0,3 | 98% |
| **ABP501-62** | 15,1 | <0,3 | 98% |
| **ABP501-63** | 6,8 | <0,3 | 96% |
| **ABP501-64** | 11,2 | <0,3 | 97% |
| **ABP501-65** | 7,3 | <0,3 | 96% |
| **ABP501-66** | 5,6 | <0,3 | 95% |
| **ABP501-67** | 3,6 | <0,3 | 92% |
| **ABP501-68** | 5,5 | <0,3 | 95% |

| **a.** | | | | | |
| --- | --- | --- | --- | --- | --- |
| ADALIMUMAB Samples | SAMPLES spiked with ABP501-Batch1 | | | | |
|  | No-inhibited* | | Inhibited** | | % of inhibition*** |
|  | µg/ml | mean | µg/ml | mean |  |
| Low | 1,3 | 1,3 | <0,3 | <0,3 | >77% |
|  | 1,3 |  | <0,3 |  |  |
| Medium | 4,8 | 5,1 | <0,3 | <0,3 | >94% |
|  | 5,4 |  | <0,3 |  |  |
| High | 18,6 | 18,5 | <0,3 | <0,3 | >98% |
|  | 18,4 |  | <0,3 |  |  |
|  |  |  |  |  |  |
| ADALIMUMAB Samples | SAMPLES spiked with ABP501-Batch2 | | | | |
|  | No-inhibited* | | Inhibited** | | % of inhibition*** |
|  | µg/ml | mean | µg/ml | mean |  |
| Low | 1,3 | 1,3 | <0,3 | <0,3 | >77% |
|  | 1,2 |  | <0,3 |  |  |
| Medium | 5,1 | 5,0 | <0,3 | <0,3 | >94% |
|  | 4,9 |  | <0,3 |  |  |
| High | 15,8 | 16,1 | <0,3 | <0,3 | >98% |
|  | 16,3 |  | <0,3 |  |  |
|  |  |  |  |  |  |
| ADALIMUMAB Samples | SAMPLES spiked with ABP501-Batch1 + ADALIMUMAB (Humira) | | | | |
|  | No-inhibited* | | Inhibited** | | % of inhibition |
|  | µg/ml | mean | µg/ml | mean |  |
| Low | 3,8 | 3,7 | <0,3 | <0,3 | >92% |
|  | 3,5 |  | <0,3 |  |  |
| Medium | 12,4 | 12,7 | <0,3 | <0,3 | >98% |
|  | 13,0 |  | <0,3 |  |  |
| High | >20 | >20 | <0,3 | <0,3 | >98% |
|  | >20 |  | <0,3 |  |  |
|  |  |  |  |  |  |
| ADALIMUMAB Samples | SAMPLES spiked with ABP501-Batch2 + ADALIMUMAB (Humira) | | | | |
|  | No-inhibited* | | Inhibited** | | % of inhibition*** |
|  | µg/ml | mean | µg/ml | mean |  |
| Low | 3,6 | 3,8 | <0,3 | <0,3 | >92% |
|  | 4,0 |  | <0,3 |  |  |
| Medium | 13,6 | 13,9 | <0,3 | <0,3 | >98% |
|  | 14,1 |  | <0,3 |  |  |
| High | >20 | >20 | <0,3 | <0,3 | >98% |
|  | >20 |  | <0,3 |  |  |

**Additional file 1: Table S2. Adalimumab assay.**

a) Biosimilar spiked samples, spiked with polyclonal antibodies directed against Adalimumab (Humira®) and Biosimilar+Humira® spiked samples, spiked with polyclonal antibodies directed against Adalimumab (Humira®)

b) Clinical samples (n> or = 30) from patients treated with adalimumab biosimilar spiked with polyclonal antibodies directed against adalimumab (Humira).

**Acceptance criteria : % of inhibition >50%**

|  |  | **ANTI-ADALIMUMAB SAMPLES DILUTED WITH BUFFER SPIKED WITH :** | | | | | |
| --- | --- | --- | --- | --- | --- | --- | --- |
| *ANTI-ADALIMUMAB CLINICAL SAMPLES* | | ADALIMUMAB (Humira) | | ABP501-Batch1 | | ABP501-Batch2 | |
| *ID* | *Anti-ADALIMUMAB (ng/ml)* | Anti-ADALIMUMAB (ng/ml) | % of inhibition* | Anti-ADALIMUMAB (ng/ml) | % of inhibition* | Anti-ADALIMUMAB (ng/ml) | % of inhibition* |
| *AADA1* | *48* | <10 | 79% | <10 | 79% | <10 | 79% |
| *AADA2* | *39* | <10 | 74% | <10 | 74% | <10 | 74% |
| *AADA3* | *43* | <10 | 77% | <10 | 77% | <10 | 77% |
| *AADA4* | *31* | <10 | 68% | <10 | 68% | <10 | 68% |
| *AADA5* | *52* | <10 | 81% | <10 | 81% | <10 | 81% |
| *AADA6* | *42* | <10 | 76% | <10 | 76% | <10 | 76% |
| *AADA7* | *49* | <10 | 80% | <10 | 80% | <10 | 80% |
| *AADA8* | *26* | <10 | 62% | <10 | 62% | <10 | 62% |
| *AADA9* | *46* | <10 | 78% | <10 | 78% | <10 | 78% |
| *AADA10* | *41* | <10 | 76% | <10 | 76% | <10 | 76% |
| *AADA11* | *58* | <10 | 83% | <10 | 83% | <10 | 83% |
| *AADA12* | *44* | <10 | 77% | <10 | 77% | <10 | 77% |
| *AADA13* | *38* | <10 | 74% | <10 | 74% | <10 | 74% |
| *AADA14* | *33* | <10 | 70% | <10 | 70% | <10 | 70% |
| *AADA15* | *49* | <10 | 80% | <10 | 80% | <10 | 80% |
| *AADA16* | *44* | <10 | 77% | <10 | 77% | <10 | 77% |
| *AADA17* | *48* | <10 | 79% | <10 | 79% | <10 | 79% |
| *AADA18* | *67* | <10 | 85% | <10 | 85% | <10 | 85% |
| *AADA19* | *53* | <10 | 81% | <10 | 81% | <10 | 81% |
| *AADA20* | *54* | <10 | 81% | <10 | 81% | <10 | 81% |
| *AADA21* | *41* | <10 | 76% | <10 | 76% | <10 | 76% |
| *AADA22* | *63* | <10 | 84% | <10 | 84% | <10 | 84% |
| *AADA23* | *31* | <10 | 68% | <10 | 68% | <10 | 68% |
| *AADA24* | *57* | <10 | 82% | <10 | 82% | <10 | 82% |
| *AADA25* | *39* | <10 | 74% | <10 | 74% | <10 | 74% |
| *AADA26* | *64* | <10 | 84% | <10 | 84% | <10 | 84% |
| *AADA27* | *54* | <10 | 81% | <10 | 81% | <10 | 81% |
| *AADA28* | *48* | <10 | 79% | <10 | 79% | <10 | 79% |
| *AADA29* | *56* | <10 | 82% | <10 | 82% | <10 | 82% |
| *AADA30* | *39* | <10 | 74% | <10 | 74% | <10 | 74% |
| *AADA31* | *43* | <10 | 77% | <10 | 77% | <10 | 77% |

**Additional file 1: Table S3. Inhibition assay.** Clinical samples with detectable level of Anti-Adalimumab antibodies were diluted with the kit’s dilution buffer previously spiked with Adalimumab (Humira® or ABP501 were added to kit’s dilution buffer in order to prepare 2 types of “ada-buffer”). Also, the clinical samples were diluted with the kit’s dilution buffer. The 3 preparations (2 preparations made with Adalimumab and 1 preparation made without Adalimumab) were incubated 60 minutes at room temperature and quantified with LISA-TRACKER Anti-Adalimumab kit (product number LTA 003, batch: 1844).

**Acceptance criteria : % of inhibition >50%**

|  |  | **DETECTION REAGENT SPIKED WITH :** | | | |
| --- | --- | --- | --- | --- | --- |
| *ANTI-ADALIMUMAB CLINICAL SAMPLES* | | ABP501-Batch1 | | ABP501-Batch2 | |
| *ID* | *Anti-ADALIMUMAB (ng/ml)* | Anti-ADALIMUMAB (ng/ml) | % of inhibition* | Anti-ADALIMUMAB (ng/ml) | % of inhibition* |
| *AADA32* | *48* | <10 | 79% | <10 | 79% |
| *AADA33* | *39* | 14 | 64% | <10 | 74% |
| *AADA34* | *32* | <10 | 69% | <10 | 69% |
| *AADA35* | *27* | <10 | 64% | <10 | 64% |
| *AADA36* | *27* | <10 | 64% | <10 | 64% |
| *AADA37* | *24* | <10 | 58% | <10 | 58% |
| *AADA38* | *37* | <10 | 73% | <10 | 73% |
| *AADA39* | *62* | <10 | 84% | <10 | 84% |
| *AADA40* | *31* | <10 | 68% | <10 | 68% |
| *AADA41* | *23* | <10 | 56% | <10 | 56% |
| *AADA42* | *36* | <10 | 72% | <10 | 72% |
| *AADA43* | *94* | <10 | 89% | <10 | 89% |
| *AADA44* | *28* | <10 | 65% | <10 | 65% |
| *AADA45* | *74* | <10 | 86% | <10 | 86% |
| *AADA46* | *35* | <10 | 71% | <10 | 71% |
| *AADA47* | *18* | <10 | 44% | <10 | 44% |
| *AADA48* | *55* | <10 | 82% | <10 | 82% |
| *AADA49* | *30* | <10 | 67% | <10 | 67% |
| *AADA50* | *14* | <10 | 30% | <10 | 30% |
| *AADA51* | *74* | <10 | 86% | <10 | 86% |
| *AADA52* | *53* | <10 | 81% | <10 | 81% |
| *AADA53* | *29* | <10 | 65% | <10 | 65% |
| *AADA54* | *64* | <10 | 84% | <10 | 84% |
| *AADA55* | *52* | <10 | 81% | <10 | 81% |
| *AADA56* | *39* | <10 | 74% | <10 | 74% |
| *AADA57* | *30* | <10 | 67% | <10 | 67% |
| *AADA58* | *64* | <10 | 84% | <10 | 84% |
| *AADA59* | *27* | <10 | 63% | <10 | 63% |
| *AADA60* | *51* | <10 | 80% | <10 | 80% |
| *AADA61* | *70* | <10 | 86% | <10 | 86% |
| *AADA62* | *25* | <10 | 60% | <10 | 60% |
| *AADA63* | *29* | <10 | 65% | <10 | 65% |
| *AADA64* | *21* | <10 | 51% | <10 | 51% |
| *AADA65* | *34* | <10 | 71% | <10 | 71% |
| *AADA66* | *59* | <10 | 83% | <10 | 83% |
| *AADA67* | *29* | <10 | 65% | <10 | 65% |
| *AADA68* | *20* | <10 | 51% | <10 | 51% |
| *AADA69* | *40* | <10 | 75% | <10 | 75% |

**Additional file 1: Table S4. Specificity detection step.** Clinical samples with detectable level of Anti-Adalimumab antibodies were quantified with LISA-TRACKER Anti-Adalimumab kit (product number: LTA 003, batch: 1849). In order to confirm the capacity of ABP501 to block antibodies directed against Humira®, detection step was performed with or without the addition of ABP501 into the detection reagent (biotinylated Humira®) used for the detection step of Anti-Adalimumab antibodies during the assay)

**Acceptance criteria : % of inhibition >50%**

|  | **ADALIMUMAB (µg/ml)** | | | | | | |
| --- | --- | --- | --- | --- | --- | --- | --- |
| **a.** | **Kit’s stability** | | | | | | |
|  | **Levels** | **Unexposed** | Acc. Criteria (+/- 20%) | | **7d +37°c** | | |
|  |  |  | target Low | target High |  |  |  |
|  | PC (c+) | **4,1** | 1,9 | 5,6 | **5,1** | | |
| **ADALIMUMAB ABP501-batch1** | Low | **1,8** | 1,4 | 2,2 | **1,6** | | |
|  | Medium | **5,3** | 4,2 | 6,4 | **6,2** | | |
|  | High | **19,6** | 15,7 | 23,5 | **20,0** | | |
| **ADALIMUMAB ABP501-batch2** | Low | **1,1** | 0,9 | 1,3 | **1,3** | | |
|  | Medium | **4,3** | 3,4 | 5,2 | **4,8** | | |
|  | High | **18,0** | 14,4 | 21,6 | **17,5** | | |
| **b.** | **Specimen’s stability** | | | | | | |
|  | **Levels** | **Unexposed** | Acc. Criteria (+/- 20%) | | **7d +4°c** | **3d RT** | **5 x f/t cycles** |
|  |  |  | target Low | target High |  |  |  |
| **ADALIMUMAB - ABP501-Batch1** | Low | **1,2** | 1,0 | 1,4 | **1,2** | **1,0** | **1,1** |
|  | Medium | **4,0** | 3,2 | 4,8 | **4,2** | **4,2** | **4,6** |
|  | High | **12,8** | 10,2 | 15,4 | **12,1** | **13,0** | **11,2** |
| **ADALIMUMAB - ABP501-Batch2** | Low | **1,2** | 1,0 | 1,4 | **1,1** | **1,1** | **1,2** |
|  | Medium | **4,3** | 3,4 | 5,2 | **4,1** | **4,5** | **4,3** |
|  | High | **12,8** | 10,2 | 15,4 | **13,7** | **11,8** | **12,3** |

**Additional file 1: Table S5. Measure of kit and sample stability.**

a) For kit’s stability, LISA-TRACKER Adalimumab kit was stored under stress thermic condition (7 days at +37°C). Then, “ABP501 spiked samples” were tested with this “stressed” kit. Results were compared to the results obtained with the unexposed kit (stored between +2°C and +8°C).

b) For specimen’s stability, spiked samples from patients treated with adalimumab biosimilar were stored in different conditions until quantification:
at -20°C (unexposed samples), 7 days between +2°C and +8°C (+4°C storage condition), 3 days between +18°C and +24°C (room temperature (RT) storage condition), and 5 freeze/thaw cycles undergone.

**Acceptance criteria : +/-20%**
